# On Position during Anæsthesia

**Published:** 1911-05-13

**Authors:** 


### Anæsthetics

### ON POSITION DURING ANÆSTHESIA.

When nitrous oxide or ethyl-chloride is to be administered in the sitting position, the anæsthetist should direct the patient to sit well back in the chair and to clasp the hands between the knees. The elbows should not be rested on the arms of the chair, nor the feet planted firmly against the foot-rest nor on the ground, as in case of stiffening occurring such support will enable him to rise out of position. It is advisable to have a strap or band to secure the legs to the chair and another passing from the back of the chair round the haunches, the fastenings of which should be such that they can be instantly undone, for, apart from rigidity, semi-voluntary struggling may occur during partial anæsthesia and much damage may be caused. The head should be supported in an upright and unconstrained position. Opportunity should be taken to bend the head and shoulders forward if there is bleeding within the mouth, nose, or throat, and this position should be maintained if re-administration is required in such cases, taking care that blood and mucus does not collect in the face-piece and obstruct breathing.

For most operations the patient should lie on the back during induction. The arms should be extended by the sides under the blanket, which should be well tucked round them. Usually one or two pillows are preferred, which may be afterwards removed if advisable. The head should be turned to one side, cough being less likely to be caused and mucus escaping more readily. When fully anæsthetised, if this position is to be maintained the arms must be secured out of the operator's way. Usually they can remain by the sides with the extended hands partly underneath the buttocks, or a length of bandage may be passed under the loins and looped round the wrists. If the table is very narrow, or for some other reason this is not convenient, the wrists should be fastened up near the shoulders by a length of bandage passed under the shoulder-blades, by lengths tying them to the table, or by safety-pins through the sleeves. The hands should not lie nor be clasped on the chest, as they are apt to be in the surgeon's way or to slip down, besides interfering with respiratory movement. The anæsthetist should see that there is no great weight of folded night clothing, blankets, macintoshes, etc., on the patient's chest. If the elbows are flexed he should remember that the radial pulses may be thereby diminished or obliterated, and do not indicate the state of the circulation.

The arms must never be allowed to hang down—pressure against the edges of the table may cause paralysis. Nor should they be over-extended with the head turned to the opposite side in operations on the breast and axilla. This again may have the effect of stretching or nipping nerve trunks injuriously. The abdominal muscles are better relaxed if the knees are flexed on a pillow and the shoulders slightly raised. A cushion under the pelvis or under the lower ribs (sometimes required) may embarrass breathing by straining the recti and throwing the abdominal viscera on the diaphragm.

A patient suffering from orthopnœa or otherwise unable to lie completely down must, of course, be anæsthetised in that position which is necessary. In some cases, *e.g.* evacuation of fluid, it may be possible to modify it by degrees later on.

With head extended over table-edge: this is seldom now used. Formerly employed with a view to prevent inhalation of blood during operations for cleft palate and for removal of adenoids, it has been found not to do so—hæmorrhage is increased, and the nose becoming blocked by coagula the blood does not run out. It is also apt to interfere with free breathing by stretching the trachea, straining all the structures of the neck, and fixing the chest. If required for bronchoscopy, etc., some support should be given to the head.

The Trendelenburg position is unsuitable for obese and florid patients and those suffering from respiratory difficulties or arterial degeneration. It is rather apt to increase lingual and laryngeal spasm and rigidity of the abdominal muscles and to cause respiratory obstruction by the tongue becoming engorged or falling against the palate. Ether, especially with a bag-inhaler, is therefore unsuitable in most cases, and fortunately there is in this position far less risk of shock, reflex or otherwise, so that chloroform or a chloroform-ether mixture may be given with more confidence. The anæsthetist should see that the knees are at the right level for flexion over the angle of the table when this is adjusted, and that the shoulder-rests are in proper position. The neck should be slightly flexed and the head supported on a pillow which may rest on the knees of the anæsthetist, who should sit on a low stool. Great care should be taken to avoid sudden raising at the conclusion of the operation, especially in anæmic and exhausted subjects.

In the lithotomy or perineal position the strap should be passed under the armpit of the side to which the head is turned. The anæsthetist must steady the head when the patient is moved down the table. Care should be taken that when the thighs are flexed no pressure is made on the abdomen by thick folds of blanket.

In lateral and latero-prone positions the breathing is hampered by the body-weight falling on the lower side of the trunk, especially when a cushion is put under the flank as in kidney operations. There may also be interference with expansion of the chest on the uppermost side, as in the case of a kidney tumour interfering with the action of the diaphragm, or in empyema. In the lateral position the uppermost arm should be supported on Braine's arm-rest, or (if this is not at hand) on a box or something similar placed on a support at the side of the table. In the latero-prone position the arm should be raised on a pillow. Care should be taken that the other arm is not crushed under the body.

